# The Pkn22 Ser/Thr kinase in *Nostoc* PCC 7120: role of FurA and NtcA regulators and transcript profiling under nitrogen starvation and oxidative stress

**DOI:** 10.1186/s12864-015-1703-1

**Published:** 2015-07-29

**Authors:** Fan Yingping, Sylvain Lemeille, Andrés González, Véronique Risoul, Yann Denis, Pierre Richaud, Otmane Lamrabet, Maria F Fillat, Cheng-Cai Zhang, Amel Latifi

**Affiliations:** Aix-Marseille University and CNRS, Laboratoire de Chimie Bactérienne - UMR7283, IMM, 31 Chemin Joseph Aiguier, 13402 Marseille cedex 20, France; Department of Microbiology and Molecular Medicine, CMU, Medical Faculty, University of Geneva, Genève, 1211 Switzerland; Departamento de Bioquímica y Biología Molecular y Celular, Universidad de Zaragoza, 50009 Zaragoza, Spain; Plate-forme Transcriptome FR3479, IMM-CNRS, Marseille, France; CEA, DSV, IBEB, SBVME, Saint-Paul-lez-Durance, F-13108 France; CNRS, UMR 7265 Biol Veget & Microbiol Environ, Saint-Paul-lez-Durance, F-13108 France; Aix Marseille Université, BVME UMR7265, Marseille, F-13284 France

**Keywords:** Cyanobacteria, *Nostoc*, Ser/Thr kinase, Oxidative stress, Nitrogen starvation, Signalling, Microarray

## Abstract

**Background:**

The filamentous cyanobacterium *Nostoc* sp. strain PCC 7120 can fix N_2_ when combined nitrogen is not available. Furthermore, it has to cope with reactive oxygen species generated as byproducts of photosynthesis and respiration. We have previously demonstrated the synthesis of Ser/Thr kinase Pkn22 as an important survival response of *Nostoc* to oxidative damage. In this study we wished to investigate the possible involvement of this kinase in signalling peroxide stress and nitrogen deprivation.

**Results:**

Quantitative RT-PCR experiments revealed that the *pkn22* gene is induced in response to peroxide stress and to combined nitrogen starvation. Electrophoretic motility assays indicated that the *pkn22* promoter is recognized by the global transcriptional regulators FurA and NtcA. Transcriptomic analysis comparing a *pkn22-*insertion mutant and the wild type strain indicated that this kinase regulates genes involved in important cellular functions such as photosynthesis, carbon metabolism and iron acquisition. Since metabolic changes may lead to oxidative stress, we investigated whether this is the case with nitrogen starvation. Our results rather invalidate this hypothesis thereby suggesting that the function of Pkn22 under nitrogen starvation is independent of its role in response to peroxide stress.

**Conclusions:**

Our analyses have permitted a more complete functional description of Ser/Thr kinase in *Nostoc*. We have decrypted the transcriptional regulation of the *pkn22* gene, and analysed the whole set of genes under the control of this kinase in response to the two environmental changes often encountered by cyanobacteria in their natural habitat: oxidative stress and nitrogen deprivation.

**Electronic supplementary material:**

The online version of this article (doi:10.1186/s12864-015-1703-1) contains supplementary material, which is available to authorized users.

## Background

Protein phosphorylation, catalysed by protein kinases, regulates a variety of activities in both prokaryotic and eukaryotic cells. It plays a critical role in cellular response to environmental stimuli by regulating gene expression and enzyme activity. Protein serine/threonine and tyrosine kinases are widespread among prokaryotes although they were identified much later than in their eukaryotic counterparts [1, 2]. The first example of Ser/Thr kinases in cyanobacteria was reported in *Nostoc* sp. PCC 7120 [3]. Complete sequencing of the *Nostoc* genome has since revealed the existence of a family of 53 putative Ser/thr and Tyr kinases, indicating their important role in the physiology of this bacterium. *Nostoc* is a filamentous cyanobacterium that, in the absence of combined nitrogen, is able to differentiate specialized cells called heterocysts for molecular nitrogen fixation [4]. NtcA, a global transcriptional regulator of the Crp (cyclic AMP receptor protein) family is necessary for the initiation of heterocyst differentiation, as well as for carbon and nitrogen metabolism in general [5]. The effector of NtcA is 2-oxoglutarate, which constitutes the molecular signal inducing cellular differentiation. The mutant strain CSE2 corresponding to an insertional mutant of *ntcA*, is unable to grow on either nitrate or dinitrogen whereas it grows on ammonium [5].

One of the most important limiting factors for cyanobacterial growth in aquatic ecosystems is iron deficiency. Cellular responses have evolved to cope efficiently with such frequently occurring iron-limited conditions. The transcriptional regulator FurA orchestrates the cellular response to iron deficiency in *Nostoc* PCC 7120 [6, 7]. A finely tuned iron homeostasis is essential since an excess on the one hand generates the hydroxyl radical, a highly reactive oxidant, through the oxidation of iron in the Fenton reaction [8]. On the other hand, in cyanobacteria, iron deficiency also generates reactive oxygen species leading to oxidative damage [9]. Moreover, FurA has been shown to regulate a number of genes important in cellular defence against oxidative stress including, among others, several genes encoding thioredoxins and the *alr3808* gene encoding the DNA stress binding protein DpsA [10].

We have previously reported that the *pkn22* (*alr2502*) gene encoding a putative Ser/Thr kinase is induced by both iron starvation and oxidative stress in *Nostoc* [11]. Our findings in the present study show that the transcription of *pkn22* is also induced under nitrogen starvation, and is under the control of FurA and NtcA. Using a microarray approach, we identified all the gene transcripts displaying a change in abundance in the *pkn22* mutant with respect to the wild type strain challenged by nitrogen starvation or peroxide stress. By comparing the transcript profiles of WT and *pkn22* mutant under these two conditions, we propose a working model for how Pkn22 functions within a signalling cascade connecting the global transcript changes in response to these two stresses.

## Results

### NtcA and FurA regulators interact with the *pkn22* promoter

**Fig. 1:**
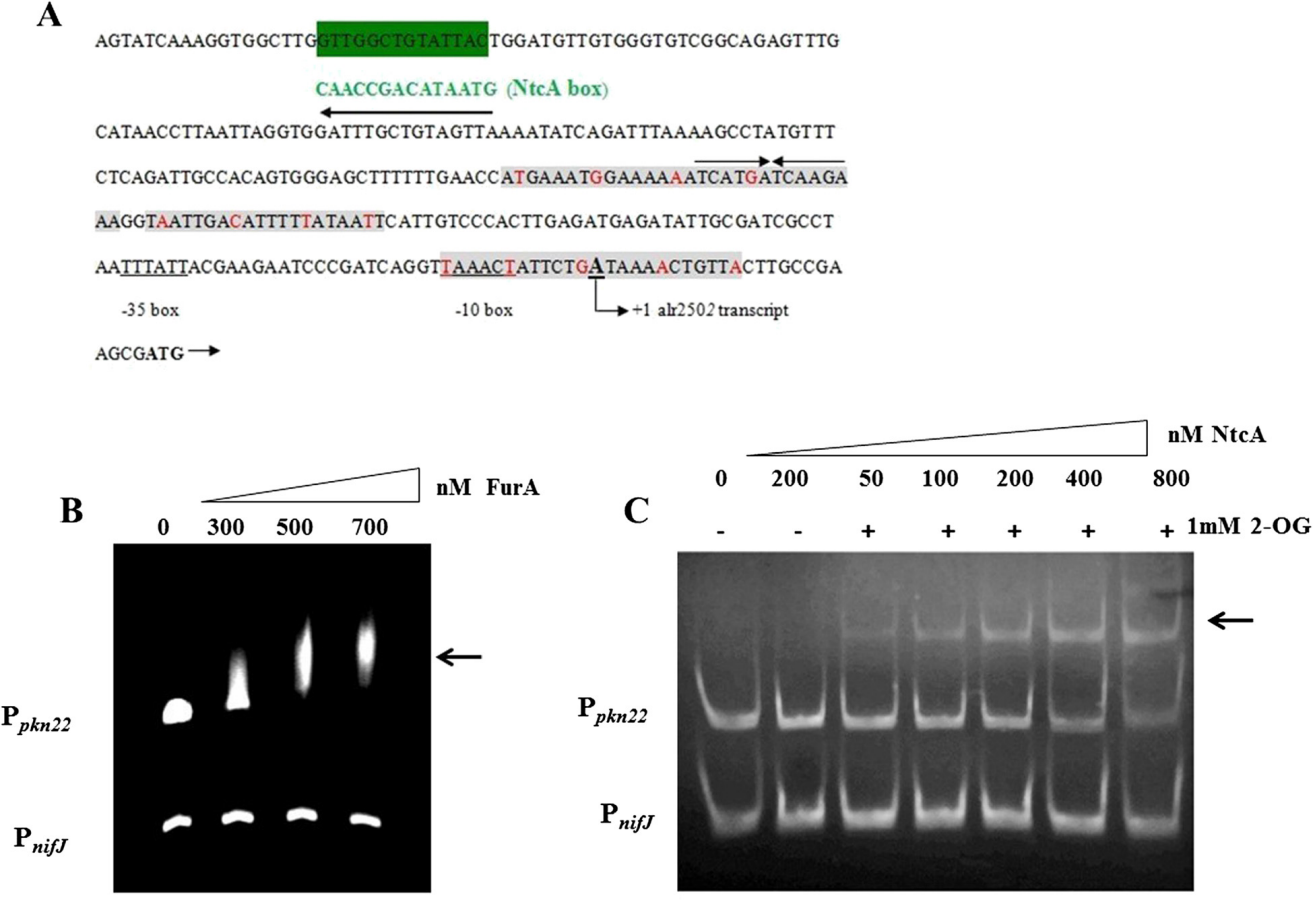
**a** In silico prediction of putative Fur and NtcA boxes in the promoter region of *alr2502* (*pkn22*). The Fur boxes are shown in red and the A/T rich region shaded in grey. The palindromic sequence corresponding to the putative NtcA box is shaded in grey. The transcription start site, deduced from Mitschke J at al, 2011 [12], is indicated by (+1). The −35 and −10 boxes are indicated. **b** EMSA showing the ability of FurA to bind *in vitro* the promoter region of *pkn22* gene. DNA fragments (100 nM) free (line1) or mixed with recombinant FurA protein at the concentrations indicated at the top of the figure in the presence of Mn^2+^ and DTT were separated on a 4 % PAGE gel. The promoter region of *nifJ* gene was used as non-specific competitor DNA at a concentration of 200 nM. **c** EMSA showing the ability of NtcA to bind *in vitro* the promoter regions of *pkn22* gene. DNA fragments free (line1) or mixed with recombinant NtcA protein at the concentrations indicated in the presence (+) or absence (−) of 1 mM 2-OG were separated on a 7 % PAGE gel. The arrows indicate the protein-DNA complexes. This experiment was repeated four times and similar results were obtained

In silico analysis of the *pkn22* promoter region (P_*pkn22*_) revealed the presence of a putative NtcA binding site (GTt-N8-TAC) centred around 250 nucleotides upstream of the transcriptional start site defined by Mitschke *et al.* [12] (Fig. 1a). It also uncovered a putative FurA binding site composed of three repeated A/T rich regions between positions - 127 and +11 relative to the +1 transcription (Fig. 1a). To investigate whether NtcA and FurA, through binding to their respective putative binding sites, directly control the transcription of the *pkn22* gene, we performed electrophoretic motility shift assays (EMSA). The results presented in Fig. 1b, c indicate that both FurA and NtcA proteins are able to bind specifically to the *pkn22* promoter *in vitro*. The interaction of NtcA with P_*pkn22*_ was dependent on the presence of 2-oxoglutatrate in the binding buffer (Fig. 1c). In the EMSA analysis the promoter of *nifJ* gene (P_*nifJ*_) served as a negative control (Fig. 1).

### Regulation of *pkn22* expression in response to environmental stimuli

**Fig. 2:**
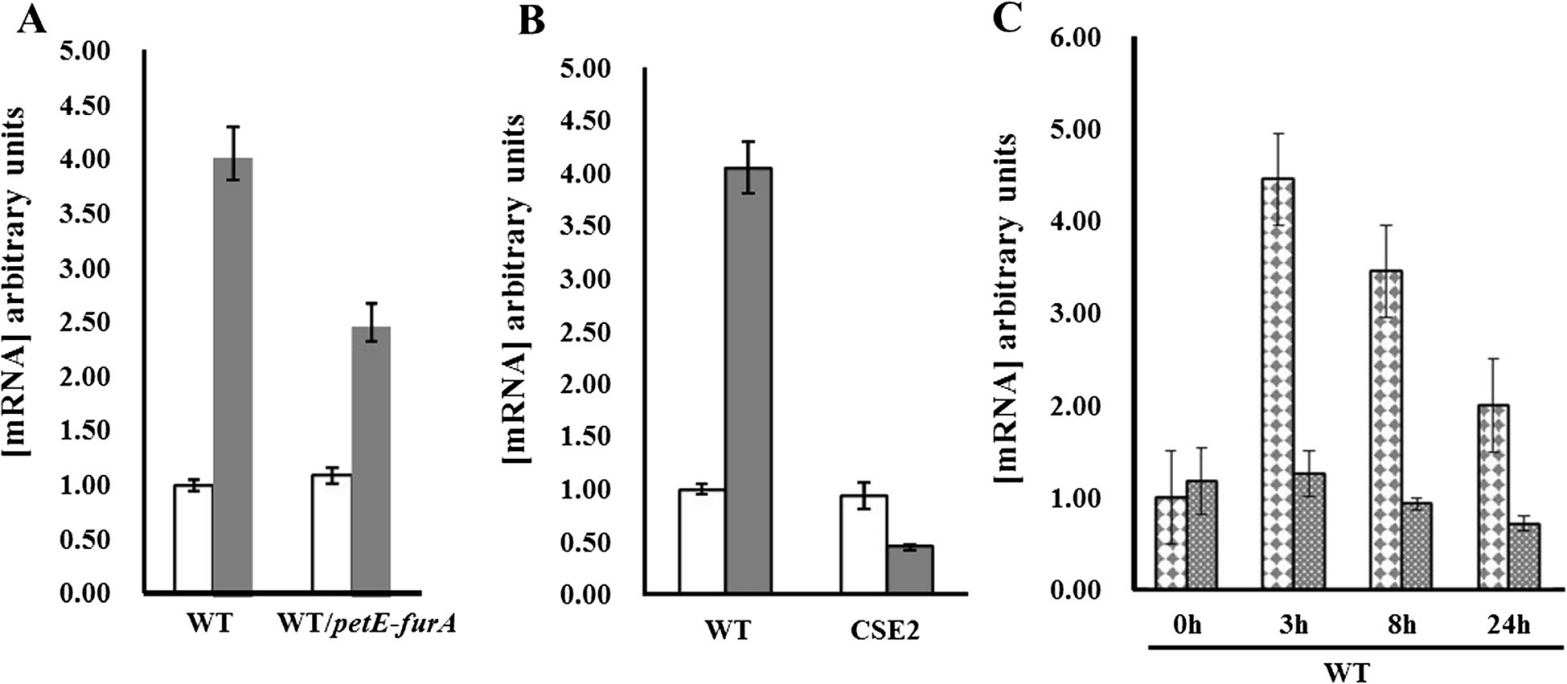
**a** qRT-PCR analysis of the *pkn22* transcripts in absence (white bars) or presence of 100 μM H_2_O_2_ during 1 h (grey bars). Data are shown as fold-change between normal and stress conditions. Each sample was measured in triplicate and the standard deviation is indicated by error bars. Values were normalized to the *rnpB* transcript. The value obtained for the condition minus H_2_O_2_ was set to 1. RNAs were extracted from *Nostoc* wild type strain (WT) or from a recombinant strain expressing the *furA* gene from the *petE* promoter (WT/*petE-furA*). **b** qRT-PCR analysis of the *pkn22* transcripts in absence (white bars) or presence (grey bars) of 100 μM H_2_O_2_ during 1 h. Data are expressed as fold-change from normal conditions. Each sample was measured in triplicate and the standard deviation is indicated by error bars. Values were normalized to the *rnpB* transcript. The value obtained for the condition minus H_2_O_2_ was set to 1. RNAs were extracted from *Nostoc* wild type strain (WT) or from the *ntcA* mutant (CSE2). **c** Quantitative RT-PCR analysis of the *pkn22* transcripts at different times (3–8 and 24 h) after nitrogen step down. RNAs were extracted from the wild type strain () or the CSE2 strain (). Data are expressed as fold-change from normal conditions. Each sample was measured in triplicate and the standard deviation is indicated by error bars. Values were normalized to the *rnpB* transcript

Our EMSA data suggest that the expression of *pkn22* is under the dual control of NtcA and FurA. We have previously reported the induction of *pkn22* gene transcription in response to oxidative stress [11]. We wished to analyse whether this control is achieved by FurA. Since the *furA* gene is essential under these conditions and therefore a *furA* mutant is not viable [13], we assessed the expression of *pkn22* by quantitative reverse transcription-polymerase chain reaction (qRT-PCR) using RNAs extracted from the *Nostoc* wild type (WT) strain or a strain overexpressing the *furA* gene (WT/*petE*-*furA*). The strains used were first incubated with or without 100 μM hydrogen peroxide (H_2_O_2_) during 1 h. The data presented in Fig. 2a show a slight decrease of 1.8 fold of *pkn22* induction in response to 1 h peroxide stress when FurA was overexpressed, suggesting that FurA might repress the *pkn22* promoter *in vivo*. We then compared the levels of *pkn22* mRNAs in response to peroxide stress between the WT strain and strain CSE2 in which the *ntcA* gene is inactivated [5]. The induction of *pkn22* expression in response to 1 h H_2_O_2_ stress was abolished in the CSE2 strain (Fig. 2b), indicating a requirement for NtcA to activate *pkn22* transcription under this condition. Since *ntcA* expression was not induced after H_2_O_2_ treatment (Additional file 1: Figure S1), we suggest that the activation of *pkn22* transcription by NtcA may rely on an allosteric modification of this regulator under such conditions.

Interestingly, the level of *pkn22* transcription increased from 3 h after nitrogen step down (Fig. 2c). Such an increase was absent in the CSE2 mutant, suggesting that the NtcA protein activated *pkn22* transcription under combined nitrogen starvation. The mechanism of action of NtcA in response to nitrogen step down has been clearly elucidated. It relies on the interaction of this transcriptional regulator with 2-oxoglutarate, which is the molecular signal of combined nitrogen starvation [14].

### *pkn22* regulon in response to peroxide stress

To address the molecular basis of the Pkn22 signalling process, we began by performing a global transcription analysis of the *pkn22* mutant compared to the WT strain under different growth conditions. The *Nostoc* whole genome microarray from Agilent was used as described previously [15]. The *pkn22* insertion mutant used in this study was obtained previously and was shown to have a growth defect under combined nitrogen starvation, when compared to the wild type strain. This mutant was also demonstrated as unable to sustain growth under oxidant conditions [9, 11]. The *pkn22* gene is the first gene of an operon which also encodes the peroxiredoxin PrxQ-A and the cysteine desulphurase OsiS [16, 17]. In order to avoid a putative polar effect of the *pkn22* mutation and to determine which genes are under the control of Pkn22, we used in our transcriptomic analysis a *pkn22* mutant strain harbouring a pRL25 plasmid bearing a wild type copy of the *pkn22* gene under the control of the *petE* promoter. The resulting recombinant strain was named WT#*pkn22*/*petE*-*pkn22.*

**Fig. 3:**
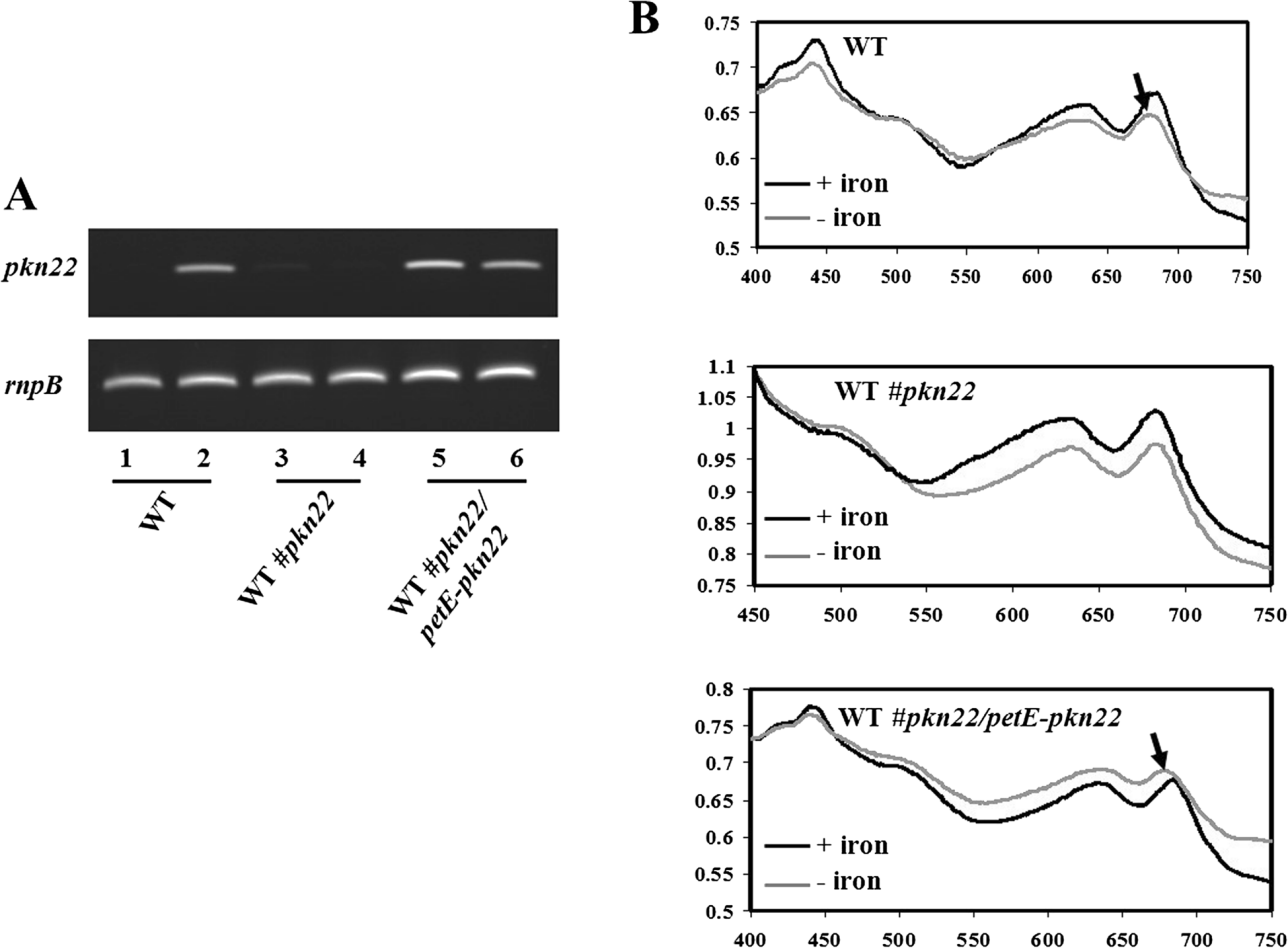
**a** Semi-quantitative RT-PCR analysis of the *pkn22* transcripts in absence (lines: 1, 3 and 5) or presence of 100 μM H_2_O_2_ (lines 2, 4 and 6). One microgram of RNA was used in each experiment. Samples were collected at the exponential phase of the PCR. The expression of the *rnpB* gene was used as a control. RNAs were extracted from *Nostoc* wild type strain (WT) or from the *pkn22* mutant (WT#*pkn22*) or from a recombinant strain expressing the *pkn22* gene from the *petE* promoter (WT*#pkn22/petE-pkn22*). **b** Absorption spectra for cell suspensions grown with (+) or without (−) iron. The arrows indicate a shift of the 680-nm chlorophyll *a* absorption peak

The results of our RT-PCR experiment presented in Fig. 3a show that whereas the induction of *pkn22* gene expression after a peroxide treatment was abolished in the *pkn22* mutant compared to the WT strain, the *pkn22* gene was expressed in the absence and presence of peroxide in the WT#*pkn22*/*petE*-*pkn22* strain which is consistent with the transcription of this gene from the *petE* promoter. Before using this strain in our transcriptomic analysis, we wanted to check if the expression of the *pkn22* gene under the *petE* promoter actually complemented the *pkn22* mutation. For this purpose, we analysed the presence of the CP43′ protein in cyanobacterial cultures challenged with iron starvation. CP43′ is a chlorophyll-binding protein conserved among cyanobacteria and produced in response to iron starvation and oxidative stress [18]. Under these conditions, CP43′ forms an antenna around photosystem I [19], an association which results in a shift of the 680-nm chlorophyll *a* absorption peak towards lower values of the spectrum [18]. In *Nostoc*, this association requires Pkn22 [11]. We exploited this requirement for Pkn22 to confirm the complementation of the *pkn22* mutation. For this, the wild type, the *pkn22* mutant and the WT#*pkn22*/*petE*-*pkn22* strains were grown in the presence or absence of iron and the absorption spectra of the cultures were analysed. While the shift of the 680-nm chlorophyll *a* absorption peak, denoting the presence of CP43′, was absent in the *pkn22* mutant spectrum, we found it in spectra of both the wild type and the WT#*pkn22*/*petE*-*pkn22* strains (Fig. 3b). This result confirms that the *pkn22* mutation was actually complemented in the WT#*pkn22*/*petE*-*pkn22* strain. Henceforth, compared to the WT strain, we considered those genes displaying a change in expression in the *pkn22* mutant and not in the WT#*pkn22*/*petE*-*pkn22* strain as being part of the *pkn22* regulon.

**Fig. 4:**
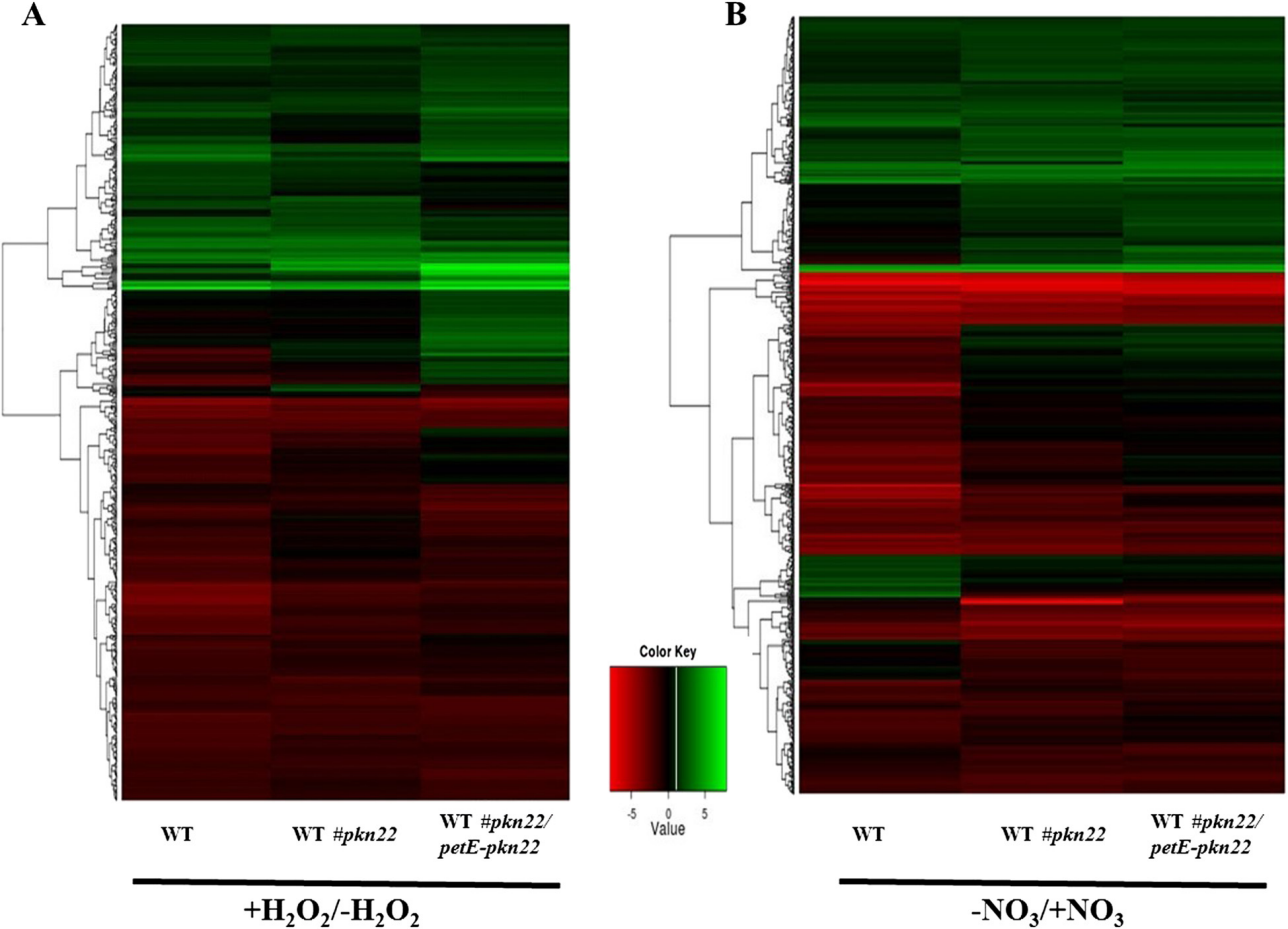
Global transcript abundance changes in response to oxidative stress (**a**) or to combined nitrogen starvation (**b**) represented by a colour-coded Heatmap. RNAs were extracted from WT strain or the *pkn22* mutant (WT#*pkn22*) or the *pkn22* mutant harbouring the pRL25 plasmid expressing the *pkn22* gene (WT*#pkn22/petE-pkn22*). Genes were hierarchically clustered according to their pattern of expression. Genes displaying a change in transcript level of at least 2-fold in at least one of the tested strains were represented. Values are expressed in log2 scale

We examined the variations in abundance of transcripts in the three *Nostoc* strains after 1 h incubation with 100 μM H_2_O_2_. Figure 4 represents a robust hierarchical clustering of the differentially expressed genes. The transcript levels of 105 genes increased with induction factors higher than 2 in the *pkn22* mutant. Among them, the transcript level of only 20 genes increased in the *pkn22* mutant and not in the WT#*pkn22*/*petE*-*pkn22* strain. In accordance with the selection criteria explained above, these genes were thus considered as being part of the *pkn22* regulon (Additional file 2: Table S2). Of the proteins encoded by these 20 identified genes, nine have unknown function and six are involved in the biosynthesis of prosthetic groups and cofactors. A third group of responsive genes included genes related to phycobilisome components (ApcA and CAB/HLIP encoding genes) and a fourth cluster contained the *ndhD* and *ndhF* genes encoding respectively the NADH dehydrogenase subunits 4 and 5. Finally, the mRNA level of the translation elongation encoding gene *tsf* also significantly increased in the *pkn22* mutant (Additional file 2: Table S2).

The transcript level of six genes specifically decreased in the *pkn22* mutant. One (*rfbD* gene) encodes a cell envelope protein and the other (*cmpC* gene) a transport and binding protein (the bicarbonate transport ATP-binding protein). The four remaining genes encode conserved hypothetical proteins according to the functional classification of Cyanobase (Additional file 2: Table S2).

### *pkn22* regulon in response to nitrogen starvation

We next obtained RNA samples from WT, *pkn22* mutant and WT#*pkn22*/*petE*-*pkn22* strains grown in the presence of nitrate or subjected to nitrogen step-down for 24 h. Microarray analysis then permitted a global transcription profiling by revealing the clustering of differentially expressed genes (Fig. 4). The transcript level of 73 genes increased specifically in the *pkn22* mutant (Additional file 3: Table S3). These genes included those encoding proteins associated with transport and binding (such as *fecD2* and *tonB* involved in iron acquisition [20]), or those required for cell envelope synthesis and cell division, as well as the Group 2 sigma factor *sigD* gene and two genes from the two-component regulatory gene family.

Of 239 genes displaying a decrease in mRNA abundance in the *pkn22* mutant, 38 were specifically responsive to the deletion of the *pkn22* gene (Additional file 3: Table S3). Among them were genes involved in translation and transcription processes, the gene encoding phycobilisome core-membrane linker protein ApcE, and *hypC* and *hypF* genes required in the synthesis and maturation of hydrogenase (Additional file 3: Table S3).

To validate the microarray data for selected genes, we performed quantitative reverse transcription assays. The RNAs used in these experiments were independent of those used for microarray studies. In particular, we analyzed the expression of 12 genes the transcript levels of which varied specifically in the *pkn22* mutant strain. In response to peroxide stress, the expression of *alr4616*, *all4791* and *all2724* genes was upregulated whereas that of *alr2879*, *alr4780* and *all0167* genes was decreased (Additional file 2: Table S2). Combined nitrogen starvation on the other hand led to an upregulation in expression of *alr0970*, *alr0657* and *alr3303* genes. Whereas the expression of *alr0020*, *all1651* and *all3181* genes was decreased (Table 1). All the qRT-PCR reactions presented greater than 80 % efficiency and in all cases results correlated well with the microarray data (Table 1).

**Table 1:** Validation of the microarray data by quantitative RT-PCR approach for selected representative genes. Fold change values are in Log2

Gene	Microarray + H_2_O_2_/-H_2_O_2_ fold change			QRT-PCR + H_2_O_2_/-H_2_O_2_ fold change		
	Wild type	*pkn* mutant	pknC strain	Wild type	*pkn* mutant	*pknC* strain
Alr4616	0.282 ± 0.36	2.321 ± 0.36	0.375 ± 0.36	0.433 ± 0.36	3.65 ± 0.41	0.136 ± 0.36
All4791	0.304 ± 0.11	2.040 ± 0.36	0.405 ± 0.36	0.398 ± 0.36	2.33 ± 0.22	0.213 ± 0.36
All2724	0.037 ± 0.41	2.428 ± 0.28	−0.151 ± 0.36	0.124 ± 0.36	2.56 ± 0.0.37	−0.104 ± 0.11
Alr2879	**−**0.541 ± 0.09	−1.871 ± 0.36	−0.331 ± 0.36	−0.446 ± 0.4	−2.024 ± 0.18	−0.254 ± 0.36
All4780	−0.680 ± 0.22	−2.437 ± 0.39	−0.902 ± 0.36	−0.789 ± 0.36	−3.012 ± 0.54	−0.489 ± 0.36
All0167	−0.661 ± 0.40	−1.784 ± 0.36	0.389 ± 0.36	−0.573 ± 0.36	−1.856 ± 0.39	0.127 ± 0.36
Gene	Microarray -N/+N fold change			QRT-PCR -N/+N fold change		
	Wild type	*pkn* mutant	*pknC* strain	Wild type	*pkn* mutant	*pknC* strain
Alr0970	−3.964 ± 0.20	1.939 ± 0.71	−2.399 ± 0,33	−2.521 ± 0,17	2.001 ± 0,22	−2.207 ± 0,19
Alr0657	−0.960 ± 0,31	2.764 ± 0,16	−1.013 ± 0,58	−0.677 ± 0,08	2.814 ± 0,36	−0.899 ± 0,15
Alr3303	0.279 ± 0,14	3.064 ± 0,22	0.420 ± 0,19	0.311 ± 0,61	2.996 ± 0,07	0.336 ± 0,29
Alr0020	−0.365 ± 0,50	−1.915 ± 0,81	−0.255 ± 0,21	−0.432 ± 0,78	−2.428 ± 0,18	−0.342 ± 0,48
All1651	0.904 ± 0,09	−2.376 ± 0,31	0.710 ± 0,39	0.872 ± 0,29	−3.010 ± 0,60	0.621 ± 0,55
All3181	2.980 ± 0,11	−1.807 ± 0,19	1.978 ± 0,46	3.017 ± 0,46	−1.995 ± 0,09	2.406 ± 0,67

### Nitrogen starvation does not generate oxidative stress or a decrease in iron

One hypothesis that could explain the involvement of Pkn22 in both nitrogen starvation and peroxide stress would be that the metabolic changes occurring after nitrogen step down unbalance the redox homeostasis thereby generating an oxidative stress. However, the transcript abundance of only one gene (all4274, *ubiH*) being common to the *pkn22* nitrogen starvation and oxidative stress regulons rather invalidates this hypothesis. Moreover, genes encoding proteins required in the defence against oxidative stress and which are highly responsive to peroxide stress [15], were not affected by nitrogen starvation (Table 2). Since iron starvation generates oxidative stress in this bacterium [9], we sought to investigate if combined nitrogen starvation could decrease the iron content of the cell. The intracellular concentration of iron in cultures grown in the presence or absence of nitrate and exposed or not to peroxide was measured as outlined in the Methods section. Surprisingly, our results clearly show that not only did nitrogen step down not lead to a decrease in the level of intracellular iron (Table 3), it actually increased it by 10-fold compared to control in the three strains analysed. While an excess of iron can generate oxidative stress via the Fenton reaction, this was not expected in the conditions tested here. Indeed, the production of reactive oxygen species would cause an increase in the expression of oxidative stress related genes which was not the case (Table 2). Altogether, these data reinforce the notion that, under our experimental conditions, combined nitrogen starvation does not generate an oxidative stress. We therefore conclude that Pkn22 is required both for the cellular response to oxidative stress and combined nitrogen starvation. It is worth noting that the iron content of the cultures submitted to both nitrogen starvation and peroxide stress was similar to that observed for nitrogen starvation alone, which suggests that the increase of the intracellular iron content is more likely a physiological response specific to combined nitrogen depletion.

**Table 2:** Comparison of the mRNA steady state of representative genes in response to peroxide stress and combined nitrogen starvation

Gene	Function	-H_2_O_2_/+H_2_O_2_ fold change	-N/+N fold change
asl4146	Sulfiredoxin SrxA	8.196	−0.793
all4145	DNA-binding stress protein	6.498	0.208
all1541	Type II Peroxiredoxin	5.809	−0.316
alr4641	2-Cysteins peroxiredoxin PrxA	5.039	−0.509
alr4642	Peroxiredoxin PrxQ	4.443	−1.048
all4003	photosystem II CP43 protein PsbC homologue	5.786	−5.678
all4000	photosystem II CP43 protein PsbC homologue	5.463	−3.351
all4002	photosystem II CP43 protein PsbC homologue	5.393	−4.714
all4001	photosystem II chlorophyll a-binding protein IsiA	5.319	−4.925
all0737	thioredoxin reductase TrxB	7.463	−0.425

**Table 3:** Measurement of intracellular iron content. *Nostoc* wild type strain was grown in presence of nitrate (BG11) or in combined nitrogen depleted medium (BG11o), or exposed to peroxide stress after nitrogen step down (BG11o H_2_O_2_) (see Experimental procedures), before the measurements were carried out. The results are the means of three independent experiments. The standard deviations are indicated

Strain	Growth conditions	Iron content (μg/g of dried cells)	
Wild type	BG11	159.13	±15.26
	BG11o	1970.02	±176.74
	BG11o + H_2_O_2_	1507.42	±190.88
WT*#pkn22*	BG11	112.69	±23.04
	BG11o	1246.31	±101.42
	BG11o + H_2_O_2_	1462.57	±165.64
WT*#pkn22/petE-pkn22*	BG11	123.89	±19.84
	BG11o	1567.24	±123.05
	BG11o + H_2_O_2_	1987.02	±167.32

## Discussion and conclusions

Our analysis of the Ser/Thr kinase Pkn22 encoding gene revealed that its transcription is induced in response to oxidative stress generated by H_2_O_2_ and to combined nitrogen starvation, and it is regulated by the global regulators FurA and NtcA.

**Fig. 5:**
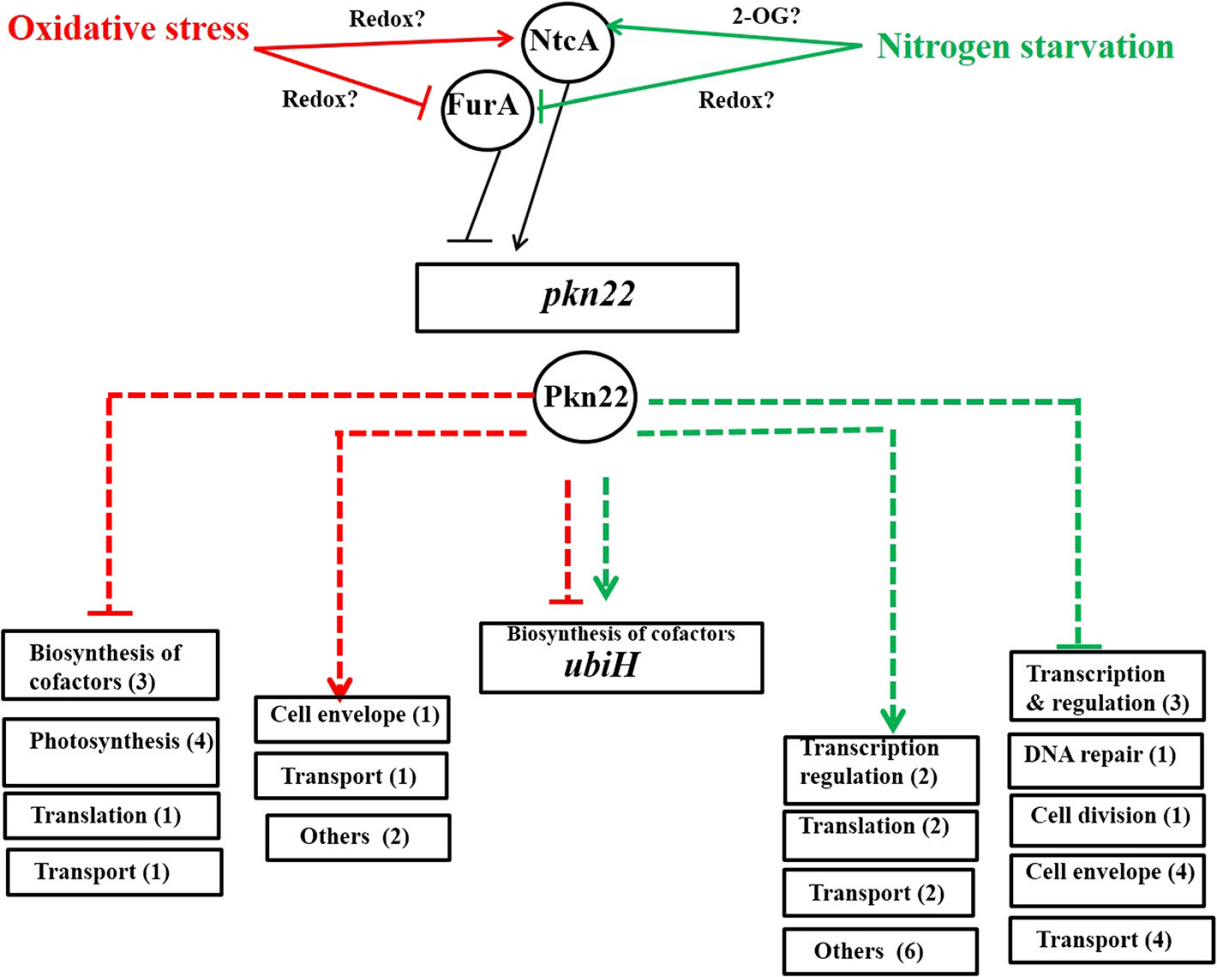
A schematic model summarizing the transcriptional regulation of *pkn22* gene, and the genes downstream for which the transcript level is under the control of the Pkn22 kinase. The red and the grey lines show the signalling cascade induced in response to peroxide stress and nitrogen starvation respectively. The dashed lines represent indirect controls. The genes are in boxes and the proteins in circles. The numbers in brackets refer to the number of genes displaying a change in level of expression in the mutant. The genes encoding proteins of unknown function and which are part of the Pkn22 regulon are not represented in this model. They are listed in Additional file 2: Table S2 and Additional file 3: Table S3

The expression of *furA* and several iron-responsive genes from *Nostoc* PCC 7120 is modulated by the master regulator of nitrogen metabolism NtcA under combined nitrogen starvation [21]. This cross-talk provides a hierarchical regulation which places NtcA at the top of the signalling cascade controlling *pkn22* expression. The mechanism that leads to FurA-derepression in response to oxidative stress remains to be elucidated. FurA protein has recently been demonstrated to be a redox-sensitive protein [22]. We propose that the redox changes that occur under conditions of peroxide stress may affect the redox state of FurA, leading to its dissociation from the promoter region of *pkn22* gene (Fig. 5).

The transcript profiling analysis of the *pkn22* mutant grown under either nitrogen starvation or peroxide stress exposure, provided evidence for the kinase playing an important role in the transduction of both signals in *Nostoc*. Indeed, the expression of many genes was significantly affected in the mutant, with the absence of the kinase having a stronger impact under combined nitrogen starvation than peroxide stress conditions. Indeed, the mRNA levels of 73 genes changed when the mutant was grown under combined nitrogen starvation versus 20 genes when it was submitted to a peroxide stress (Additional file 2: Table S2 and Additional file 3: Table S3, Fig. 5). Under peroxide stress conditions, Pkn22 regulates genes encoding proteins involved in the uptake of inorganic carbon such as CmpC or subunits of the NADH dehydrogenase complex. Pkn22 was initially identified as being necessary for the association of CP43′ with the photosynthetic machinery under oxidative stress [11]. Such modulation of photosynthesis activity upon exposure to a highly oxidative environment is important for survival of photosynthetic organisms and makes Pkn22 an important element in their cellular response to this stress. In addition, the mRNA level of a large number of genes encoding proteins of unknown function varied in both conditions in the *pkn22* mutant, suggesting an even greater role of this kinase in the signalling process of *Nostoc* than that discussed here.

We recently reported the transcriptomic profiling of *Nostoc* in response to peroxide stress [23]. The data published in this study highlighted that the PerR repressor as the major regulator of the peroxide stress stimulon. It was found to directly control the transcription of genes encoding antioxidant enzymes, such as peroxidases. It was not surprising therefore to not find such genes in the *pkn22* regulon. While we know that the signalling cascade of peroxide stress involves more than one regulator [23], what appears now clear is that PerR must be at the top of this cascade whereas Pkn22, and other transcriptional regulators most probably act downstream of PerR.

Increasing amounts of data support the existence of an overlap between oxidative stress and nitrogen regulatory networks in cyanobacteria. Enzymes involved in the reduction of oxygen or its reactive intermediates have been found produced under limited nitrogen regime in several cyanobacteria. Nitrogen limitation led to an increased synthesis of 2-Cys peroxiredoxin (2-Cys Prx) in *Synechococcus* PCC 7942 [24], and the removal of nitrate from the growth medium induced the expression of *prx* gene encoding 2 Cys-Prx in both *Synechocystis* PCC 6803 and *Synechococcus* PCC 7942 [25, 24, 23, 22]. It was thus tempting to postulate that combined nitrogen starvation generates oxidative stress which in turn triggers the expression of the *pkn22* gene. The experiments we performed to analyse this hypothesis, however, revealed that the expression of genes known to be highly induced in response to oxidative stress do not vary under nitrogen starvation (Table 2). While our results do not allow complete exclusion of the redox state of the cell being unbalanced upon forced adaptation to a new nitrogen regime, they do indicate that these cells were not transducing signals of strong oxidative stress. Consequently, *pkn22* induction in response to nitrogen starvation would appear to be independent of that from peroxide stress stimuli. This kinase can thus be considered as a regulator connecting these two signalling pathways, as suggested in the working model presented in Fig. 5.

The challenge faced by future investigations will be to elucidate the molecular mechanism by which Pkn22 transduces the nitrogen availability and the oxidation level of the cell to its targets. The most intriguing questions left unanswered are which proteins are phosphorylated and where they are situated within the regulatory pathways that have emerged from this study.

## Methods

### Bacterial strains and growth conditions

*Nostoc* sp. PCC 7120 was grown in BG11 medium or in BG11 medium without nitrate (BG11o). The cultures were grown at 30 °C in air under continuous illumination (40 μE m^−2^ s^−1^). Cyanobacterial growth was monitored by measuring the absorbance at 750 nm (OD 750). For the CSE2 mutant, nitrate was replaced by ammonium (NH_4_^+^) in the BG11 medium composition. To determine the effect of oxidative stress conditions on gene expression, cells were grown in BG11 medium until the late logarithmic phase (OD750 = 0.6) before 100 μM hydrogen peroxide was added to one-half of the culture and the cells harvested after 1 h.

### Construction of the *pkn22* and the *furA* overexpressing strains

For the overexpression of *pkn22* and *furA* genes, the entire coding region of alr2505 and alr1691 were amplified by PCR using the *pkn22* forward and reverse primers and the *furA* forward and reverse primers respectively (Additional file 4: Table S1). The amplified fragments were cloned between the NdeI and EcoRI sites downstream of the *petE* promoter in the pRL25c*petE* vector [26]. The resulting plasmids (named pRL*pkn22* and pRL*furA*) were conjugated into *Nostoc*, and exoconjugants were selected with 50 μg/ml of neomycin.

### Elemental iron content analysis

*Nostoc* cultures were grown in BG11 or in BG11o and incubated or not with 100 μM H_2_O_2_ during 1 h. The samples were mineralized overnight at 80 °C with 300 μL of ultrapure 70 % nitric acid (JT Baker) and diluted to 6 mL (final volume) in ultrapure water. Iron concentrations were measured by ICP-OES using a Vista MPX spectrometer (Agilent-Varian). Iron content was determined using a curve established with certified ICP grade standards. The measurement of each strain in each condition was performed in duplicate.

### DNA microarray construction

A whole-genome microarray comprising probes covering all the ORFs present on the chromosome and the six plasmids (Alpha, Beta, Gamma, Delta, Epsilon and Zeta) of *Nostoc,* was designed as described previously [15].

### DNA microarray experiments

RNA was extracted as previously described [11]. Chromosomal DNA was removed by treating RNA preparations with 1 μl of DNAse (at 2U/μl) (Ambion) for 1 h at 37 °C. The concentration of RNA was determined spectrophotometrically.

The cDNAs were produced using the Promega ChipShot direct labelling and clean-up system kit that ensures the incorporation of the cyanine 5 and cyanine 3 dyes during the DNA synthesis. This experiment was performed following the manufacturer’s instructions (Promega).

The hybridization and washing steps were performed using the gene expression hybridization kit and wash pack (Agilent) according to the manufacturer’s specifications. Axon GenePix 4400A (MDS Analytical technologies) was used for scanning the slides.

Experimental design, data acquisition and statistical analysis: wild type strain challenged by nitrogen starvation or treated by H_2_O_2_ (see above) was used for total RNA extraction and cDNA synthesis. For each strain, total RNA was extracted from four independent cultures for each condition (H_2_O_2_ treatment, iron starvation, or control). RNA samples to be compared were converted to cDNA, fluorescently labelled with Cy3 or Cy5 in direct (Cy3-Cy5) and dye swap (Cy5-Cy3) labelling reactions to correct for dye-dependent variation of labelling efficiency, and cohybridized on the slides. Transcriptome comparisons were performed between treated and control strains. Data are represented in Log2 values. Statistical significance of the differences in mean fluorescence intensity for each gene in the cDNA samples compared was determined by an unpaired 2-tailed Student’s *t* test. Genes were considered differentially expressed when *P* values were ≤0.001 and the relative expression was equal to or exceeded a 2-fold change on a Log2 scale (which, on a linear scale, corresponds to variations ≥4 for induced genes and ≤0.5 for repressed genes). The microarray data are presented in Additional file 5: Table S4.

### qRT-PCR

Reverse transcription: for each reaction, 1 μl of random hexamer primers (Invitrogen) and 500 ng of total RNA were denaturated at 95 °C and chilled quickly on ice. A mix consisting of 4 μl of 5x buffer, 1 μl of RNase Inhibitor (Invitrogen), 1 μl of 5 mM dNTP and 1 μl of MMLV reverse transcriptase enzyme (200U/μl, Invitrogen) was added in a total volume of 20 μl, followed by 1 h of incubation at 45 °C.

PCR conditions were identical for all reactions. The 15 μl-reaction mixture consisted of 1x GoTaq qPCR Master Mix (Promega), 0.75 μl of SYBR Green I Dye (Roche), and 500 nM final concentration of each primer. The cDNA resulting from reverse transcription was diluted 25X and used as template. PCR amplifications were carried out in CFX96 qPCR System (BioRad) as described previously [15]. The primers used in the quantitative-PCR experiments are listed in Additional file 4: Table S1.

All measurements were carried out in triplicate. The data were analysed using Software Bio-Rad CFX manager 3.0 (BioRad), and the delta Ct method. Only reactions with over 80 % efficiency were considered.

### EMSA

The promoter region of the *pkn22* gene was obtained by PCR using pkn22 RT-forward and pkn22 RT-reverse primers (Additional file 4: Table S1). The reverse primer was modified at its 3′ end by adding the cyanine 5 dye. FurA protein, purified as described previously [27, 28], was incubated with the promoter fragments (100 nM) in a buffer containing 10 mM Tris (pH 7.5), 40 mM potassium chloride, 0.1 mg/ml bovine serum albumin, 5 % glycerol, 1 mM manganese chloride (Mn^2+^), 1 mM DTT and 50 μg/ml of salmon sperm DNA. The promoter of *nifJ* gene was used in each experiment to ensure the specificity of the *pkn22*-promoter interaction. NtcA protein was purified as described previously [29]. Binding assays with the *pkn22* promoter were performed using purified NtcA protein at the concentrations indicated in Fig. 1 and target DNA (100 nM) in buffer containing 0.1 μg μl^−1^ of salmon sperm DNA, 0.25 μg μl^−1^ of bovine serum albumin, 8 % glycerol, 12 mM HEPES–NaOH (pH 8), 4 mM Tris–HCl (pH 8), 60 mM KCl, and 1 mM dithiothreitol [30].

The reactions were incubated in the dark at 30 °C during 30 min. They were then separated on 7 % native polyacrylamide gels. Electrophoresis results were visualized using a Fujifilm FLA5100 phosphoimager (Fuji).

### Availability of supporting data

All the supporting data are included as additional files.

## Additional files

Additional file 1: Figure S1. qRT-PCR analysis of the *ntcA* transcripts in absence or presence of 100 μM H_2_O_2_ during 1 h. Data are expressed as fold-change between normal and stress conditions. Each sample was measured in triplicate and the standard deviation is indicated by error bars. Values were normalized to the *rnpB* transcript. RNAs were extracted from *Nostoc* wild type strain. Additional file 2: Table S2. ᅟ Additional file 3: Table S3. ᅟ Additional file 4: Table S1. The sequence of the primers used in this study. Additional file 5: Table S4. Microarray data obtained in this study. **a**: Wild type +/− H_2_O_2_. **b**: *pkn22* mutant +/− H_2_O_2_. **c**: WT#*pkn22 petE-pkn22* strain +/− H_2_O_2_. **d**: Wild type −/+ N. **e**: *pkn22* mutant −/+ N. **f**: WT#*pkn22 petE-pkn22* strain −/+ N. **Gene ID**: gene name. **Median**: the average of four independent values obtained. **SD**: standard variation. The data were considered “valid” only if results were obtained for three of the four slides tested.

## Abbreviations

EMSA: Electrophoretic motility shift assays.
qRT-PCR: Quantitative reverse transcription-polymerase chain reaction
