# Supplementary figures and images for: The Pkn22 Ser/Thr kinase in Nostoc PCC 7120: role of FurA and NtcA regulators and transcript profiling under nitrogen starvation and oxidative stress
Source: BMC Genomics. 2015 Jul 29;16(1):557. doi: 10.1186/s12864-015-1703-1 (PMC4518582; doi:10.1186/s12864-015-1703-1)

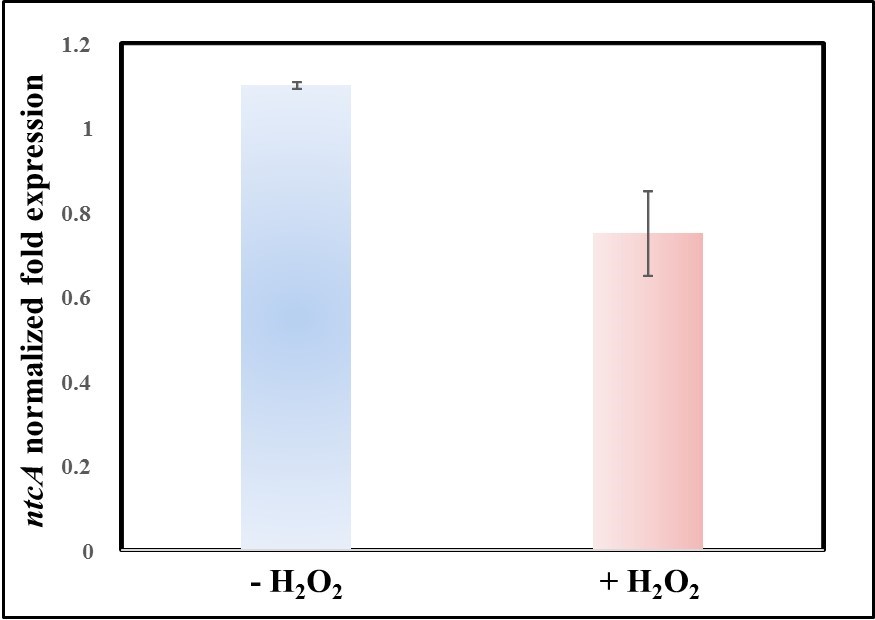

Supplement: Additional file 1: Figure S1. — qRT-PCR analysis of the ntcA transcripts in absence or presence of 100 μM H2O2 during 1 h. Data are expressed as fold-change between normal and stress conditions. Each sample was measured in triplicate and the standard deviation is indicated by error bars. Values were normalized to the rnpB transcript. RNAs were extracted from Nostoc wild type strain. [file 12864_2015_1703_MOESM1_ESM.jpg]
