# Supplementary material for: The Pkn22 Ser/Thr kinase in Nostoc PCC 7120: role of FurA and NtcA regulators and transcript profiling under nitrogen starvation and oxidative stress
Source: BMC Genomics. 2015 Jul 29;16(1):557. doi: 10.1186/s12864-015-1703-1 (PMC4518582; doi:10.1186/s12864-015-1703-1)
Supplement: Additional file 2: Table S2. — ᅟ [file 12864_2015_1703_MOESM2_ESM.docx]

Additional file 2: Table S2

| **Cyanobase ORF annotation** | **Gene** | Function | **-H_2_O_2_/+H_2_O_2_ fold change** |
| --- | --- | --- | --- |
| **Increased mRNAs** |  |  |  |
| **Biosynthesis of cofactors, prosthetic groups, and carriers** |  |  |  |
| alr4616 |  | Similar to ferrochelatase | 2.321 |
| alr3553 |  | 4-hydroxybenzoate-octaprenyl transferase | 2.149 |
| all4274 | *ubiH* | Probable 2-octaprenyl-6-methoxyphenol 4-monoxygenase; UbiH | 1.835 |
| alr0096 | *sds* | solanesyl diphosphate synthase; Sds | 1.625 |
| all4721 | *folE* | GTP cyclohydrolase I | 1.600 |
| **Transport and binding proteins** |  |  |  |
| all0126 | *cycA* | sulfate ABC transporter; ATP-binding protein CysA | 2.169 |
| **Photosynthesis and respiration** |  |  |  |
| alr3956 | *ndhF* | NADH dehydrogenase subunit 5 | 1.788 |
| all0450 | *apcA2* | Allophycocyanin alpha subunit ApcA | 1.731 |
| alr5050 | *ndhD* | NADH dehydrogenase subunit 4 | 1.643 |
| **Other categories** |  |  |  |
| asl0449 |  | CAB/ELIP/HLIP-related protein | 1.820 |
| **Translation** |  |  |  |
| all4791 | *tsf* | Translation elongation factor Ts | 2.040 |
| **Conserved hypothetical protein** |  |  |  |
| all2724 |  | Hypothetical protein | 2.428 |
| alr2201 |  | Hypothetical protein | 2.362 |
| asr1328 |  | Hypothetical protein | 2.140 |
| all1225 |  | Hypothetical protein | 1.627 |
| alr1196 |  | Hypothetical protein | 1.618 |
| asr3294 |  | Hypothetical protein | 1.617 |
| **No similarity** |  |  |  |
| alr0720 |  | Unknown protein | 1.912 |
| asl0448 |  | Unknown protein | 1.611 |
| alr4693 |  | Unknown protein | 1.603 |
| **Decreased mRNAs** |  |  |  |
| **Cell envelope** |  |  |  |
| alr4490 | *rfbD* | dTDP-6-deoxy-L-mannose-dehydrogenase | -1.614 |
| **Transport and binding proteins** |  |  |  |
| alr2879 | *cmpC* | Bicarbonate transport ATP-binding protein CmpC | -1.871 |
| **Other categories** |  |  |  |
| all0167 | *mts* | Maltooligosyltrehalose synthase | -1.784 |
| all4780 |  | Probable monooxygenase | -2.437 |
| **Conserved hypothetical protein** |  |  |  |
| all0443 |  | Hypothetical protein | -1.590 |
| **No similarity** |  |  |  |
| asl4565 |  | Unknown protein | -1.869 |
