# Supplementary material for: The Pkn22 Ser/Thr kinase in Nostoc PCC 7120: role of FurA and NtcA regulators and transcript profiling under nitrogen starvation and oxidative stress
Source: BMC Genomics. 2015 Jul 29;16(1):557. doi: 10.1186/s12864-015-1703-1 (PMC4518582; doi:10.1186/s12864-015-1703-1)
Supplement: Additional file 3: Table S3. — ᅟ [file 12864_2015_1703_MOESM3_ESM.docx]

Additional file 3: Table S3

| **Cyanobase ORF annotation** | **Gene** | **Function** | **Growth-N/+N fold change** |
| --- | --- | --- | --- |
| **Increased mRNAs** |  |  |  |
| **Transport and binding proteins** |  |  |  |
| alr0970 |  | ABC transporter ATP-binding protein | 1.939 |
| alr3240 | *fecD2* | Ferrichrome ABC transporter, permease protein | 1.752 |
| all5043 |  | Polyamine ABC transporter, polyamine-binding protein | 1.745 |
| alr0141 |  | Oligopeptide ABC transporter, permease protein | 1.623 |
| **Cell envelope** |  |  |  |
| alr0657 | *rfbB* | dTDP-glucose 4-6-dehydratase; RfbB | 2.764 |
| all4430 |  | Probable polysaccharide biosynthesis protein | 1.878 |
| all4826 | *wcaG* | dTDP-glucose dehydratase | 1.841 |
| alr4060 |  | Probable alginate O-acetylation protein | 1.762 |
| **Regulatory functions** |  |  |  |
| alr5251 |  | Two-component response regulator | 2.143 |
| alr1231 |  | Two-component hybrid sensor and regulator | 1.649 |
| **Fatty acid, phospholipid and sterol metabolism** |  |  |  |
| all2102 | *ama* | N-acyl-L-amino acid amidohydrolase | 1.841 |
| **DNA replication, recombination, and repair** |  |  |  |
| all3632 | *avalM* | Site-specific DNA-methyltransferase (cytosine-specific) | 1.747 |
| **Transcription** |  |  |  |
| alr3810 | *sigD* | Group 2 sigma 70-type sigma factor D | 1.860 |
| **Cellular processes** |  |  |  |
| alr3858 | *ftsZ* | Cell division protein FtsZ | 1.595 |
| **Biosynthesis of cofactors, prosthetic groups, and carriers** |  |  |  |
| all4274 | *ubiH* | Probable 2-octaprenyl-6-methoxyphenol 4-monoxygenase; UbiH | 1.740 |
| **Translation** |  |  |  |
| all4590 |  | Protease IV | 1.612 |
| **Purines, pyrimidines, nucleosides, and nucleotides** |  |  |  |
| all4607 | *gcvP* | Glycine cleavage system protein P | 2.143 |
| **Other categories** |  |  |  |
| all4828 | *rfbD* | GDP-D-mannose dehydratase | 2.466 |
| asl4827 |  | GDP-D-mannose dehydratase | 2.318 |
| alr1668 |  | Putative glycosyltransferase | 2.284 |
| alr5034 |  | Methanol dehydrogenase regulatory protein MorR homolog | 1.889 |
| all0919 |  | Probable glycosyltransferase | 1.836 |
| alr4168 |  | Probable glycosyltransferase | 1.764 |
| all4426 |  | Probable glycosyltransferase | 1.752 |
| alr3057 |  | Probable glycosyltransferase | 1.606 |
| all1766 |  | Probable glycosyl transferase | 1.587 |
| **Conserved hypothetical protein** |  |  |  |
| alr3303 |  | Hypothetical protein | 3.064 |
| alr0971 |  | Hypothetical protein | 2.253 |
| asl3784 |  | Hypothetical protein | 2.150 |
| all7011 |  | Hypothetical protein | 2.147 |
| all1871 |  | Hypothetical protein | 1.982 |
| alr3361 |  | Hypothetical protein | 1.965 |
| alr3304 |  | Hypothetical protein | 1.937 |
| asl1262 |  | Hypothetical protein | 1.933 |
| all5040 |  | Hypothetical protein | 1.909 |
| all2750 |  | Hypothetical protein | 1.862 |
| alr2940 |  | Hypothetical protein | 1.751 |
| alr3833 |  | Hypothetical protein | 1.738 |
| all1251 |  | Hypothetical protein | 1.727 |
| all2040 |  | Hypothetical protein | 1.683 |
| alr0622 |  | Hypothetical protein | 1.668 |
| alr3714 |  | Hypothetical protein | 1.663 |
| alr3730 |  | Hypothetical protein | 1.628 |
| all0591 |  | Hypothetical protein | 1.601 |
| all0384 |  | Hypothetical protein | 1.598 |
| **No similarity** |  |  |  |
| alr3302 |  | Unknown protein | 3.209 |
| alr3554 |  | Unknown protein | 3.053 |
| alr3301 |  | Unknown protein | 2.923 |
| all4427 |  | Similar to phytanoyl-CoA hydroxylase | 2.357 |
| asl4328 |  | Unknown protein | 2.217 |
| asl5041 |  | Unknown protein | 2.205 |
| all4744 |  | Unknown protein | 2.176 |
| all1708 |  | Unknown protein | 2.122 |
| alr3816 |  | Unknown protein | 2.073 |
| alr0722 |  | Unknown protein | 1.968 |
| alr2117 |  | Unknown protein | 1.911 |
| all4578 |  | Unknown protein | 1.908 |
| alr1851 |  | Unknown protein | 1.903 |
| alr4863 |  | Unknown protein | 1.800 |
| all4982 |  | Unknown protein | 1.786 |
| alr4505 |  | Unknown protein | 1.755 |
| asl7051 |  | Unknown protein | 1.736 |
| all0565 |  | Unknown protein | 1.731 |
| alr0150 |  | Unknown protein | 1.705 |
| all5244 |  | Unknown protein | 1.686 |
| all3034 |  | Unknown protein | 1.676 |
| all5245 |  | Unknown protein | 1.673 |
| all4083 |  | Unknown protein | 1.660 |
| all5036 | *tonB3* | Unknown protein | 1.658 |
| all1875 |  | Unknown protein | 1.648 |
| all7198 |  | Unknown protein | 1.633 |
| all8507 |  | Unknown protein | 1.624 |
| alr1045 |  | Unknown protein | 1.598 |
| **Decreased mRNAs** |  |  |  |
| **Transport and binding proteins** |  |  |  |
| all7122 |  | Probable transporter | -1.591 |
| all7592 |  | Cation transporting ATPase | -1.643 |
|  |  |  |  |
| **Photosynthesis and respiration** |  |  |  |
| alr0020 | *apcE* | Phycobilisome core-membrane linker protein ApcE | -1.915 |
| **Regulatory functions** |  |  |  |
| alr3761 |  | Two-component hybrid sensor and regulator | -1.897 |
| all1651 |  | Transcriptional regulator | -2.376 |
| **Purines,pyrimidines,nucleosides,andnucleotides** |  |  |  |
| all3180 |  | Similar to adenyl atecyclase | -1.807 |
| **Translation** |  |  |  |
| all4215 | *rpl3* | 50S ribosomal protein L17 | -1.665 |
| all4208 | *rpl16* | 50S ribosomal protein L16 | -1.673 |
| **Other categories** |  |  |  |
| alr7386 | alr7386 | Transposase | -1.612 |
| all0569 |  | Iron-sulfur cluster binding protein homolog | -1.658 |
| asr0695 | *hypC* | Hydrogenase expression/formation protein HypC | -1.882 |
| all0111 |  | Esterase | -2.143 |
| alr0694 | *hypF* | Hydrogenase maturation protein HypF | -2.193 |
| **Conserved hypothetical protein** |  |  |  |
| alr1346 |  | Hypothetical protein | -1.589 |
| all4540 |  | Hypothetical protein | -1.602 |
| alr2616 |  | Hypothetical protein | -1.644 |
| all4041 |  | Hypothetical protein | -1.645 |
| asl5170 |  | Hypothetical protein | -1.738 |
| asl4146 | *srxA* | Hypothetical protein | -2.020 |
| asl4765 |  | Hypothetical protein | -2.023 |
| alr7524 |  | Hypothetical protein | -2.178 |
| all7594 |  | Hypothetical protein | -2.181 |
| all3181 |  | Hypothetical protein | -2.871 |
| **No similarity** |  |  |  |
| asr3266 |  | Unknown protein | -1.593 |
| asl4753 |  | Unknown protein | -1.597 |
| asr0701 |  | Unknown protein | -1.626 |
| alr5330 |  | Unknown protein | -1.627 |
| asr1195 | *asr1195* | Unknown protein | -1.732 |
| all1273 | *all1273* | Unknown protein | -1.860 |
| asr0636 |  | Unknown protein | -1.895 |
| all3186 |  | Unknown protein | -1.896 |
| asl0206 |  | Unknown protein | -1.922 |
| asl1274 |  | Unknown protein | -2.092 |
| all4254 |  | Unknown protein | -2.111 |
| all5171 |  | Unknown protein | -2.125 |
| all5281 |  | Unknown protein | -2.207 |
| alr1184 |  | Unknown protein | -2.316 |
| asl7593 |  | Unknown protein | -2.371 |
