# Supplementary material for: The Pkn22 Ser/Thr kinase in Nostoc PCC 7120: role of FurA and NtcA regulators and transcript profiling under nitrogen starvation and oxidative stress
Source: BMC Genomics. 2015 Jul 29;16(1):557. doi: 10.1186/s12864-015-1703-1 (PMC4518582; doi:10.1186/s12864-015-1703-1)
Supplement: Additional file 4: Table S1. — The sequence of the primers used in this study. [file 12864_2015_1703_MOESM4_ESM.docx]

Additional file 4: Table S1:

The sequence of the primers used in this study.

| Primer | Direction | Primer Sequence(5’-3’) | Feature |
| --- | --- | --- | --- |
| *rnpB* | Forward | AGGGAGAGAGTAGGCGTTGC | qRT-PCR |
|  | Reverse | GGTTTACCGAGCCAGTACCTC T |  |
| *pkn22-RT* | Forward | ATTTCCGCAGGTTACA CACC | qRT-PCR |
| *ntcA-RT* | Reverse  Forward  Reverse | CTGGAGAGTAGCCCATCACG  CGTGACACAAGATAAGGCCC  CTGGAAAGTTTCACAGCCCC | qRT-PCR |
| *pkn22prom* | Forward | ATATCAAGAGTGTCTTCGTTAAT | EMSA |
|  | Reverse | GTTTTTGTGGTTTTGAGC |  |
| *pkn22Competitor* | Forward | ACTGGCGTGATGGGCTACTC | EMSA |
|  | Reverse | CGTCGTCTCGTCCATCCTTT |  |
| *furA* | Forward | GGATCCATGACTGTCTACACAAATACTTCGCT | *furA* overexpression |
|  | Reverse | CTCGAGCTAAAGTGGCATGAGCGCACG |  |
| *pkn22* | Forward | CCATATGATGAGCCCTCTGCATA | *pkn22*complemention |
|  | Reverse | CGAATTCCTACTCTACATTGCCG |  |
